# Why it is easier to date Quaternary extinctions than human colonizations under conditions of overkill

**DOI:** 10.1017/ext.2026.10017

**Published:** 2026-07-21

**Authors:** Todd A. Surovell

**Affiliations:** Department of Anthropology, University of Wyoming, USA

**Keywords:** Quaternary extinction, human colonization, predator–prey dynamics, taphonomic bias

## Abstract

Faunal extinctions correlate with human colonization worldwide during the Quaternary, yet the extent to which human hunting drove these losses remains debated. Resolving these debates depends on accurately dating both human arrival and animal extinction events. Herein I present a simulated overkill scenario modeling interaction between a founding human population and a single prey species. The simulation evaluates expected age offsets, the differences between the actual and observed dates, for extinction and colonization as functions of sample size and human population growth rate. Results show that when extinction is driven by human hunting, age offsets for extinction are expected to be smaller than those for colonization. This difference in age offset is expected because overkill produces a rapid demographic collapse of prey populations and taphonomic processes concentrate late-surviving individuals in the fossil record. In contrast, colonization dates are more offset because human populations are smallest immediately after arrival, and archaeological rarity is compounded by taphonomic effects. Although colonization age offsets are consistently larger than extinction age offsets across sample sizes, they are not extreme, such that archaeological sites should be detectable within five centuries of initial human settlement under modest rates of human population growth.

## Impact statement

As humans colonized the globe, faunal extinctions followed, particularly among large-bodied animals. Nonetheless, for many colonization and extinction events on islands and continents, considerable debate persists regarding the timing of human arrival and animal extinction, as well as the extent to which overhunting contributed to prey extirpation. Accurately dating both colonization and extinction events is essential to resolving these debates. Doing so is inherently challenging. It requires identifying the earliest archaeological evidence of human presence and the most recent occurrences of extinct taxa in a region, both of which are expected to be rare in the archaeological and fossil records. In this study, I simulate the overkill of a prey species by a colonizing human population and use the resulting population dynamics to generate expected archaeological and paleontological records. I then repeatedly sample these simulated records to estimate expected age offsets for both human arrival and prey extinction. The simulations show that if overkill produces a rapid population collapse of animal populations, remains dating close to the true extinction window can be identified with relatively small numbers of specimens. In contrast, detecting the earliest archaeological sites is substantially more difficult, because they occur when human populations are small and leave a sparse material signature. With limited samples of early archaeological sites and late-occurring extinct taxa, extinction may even appear to precede human arrival. The results suggest that debates over the timing and causes of Quaternary extinctions and human colonization are empirically resolvable, provided sufficiently large samples of dates are obtained. These findings therefore have wide relevance for archaeologists and paleontologists working around the world.

## Introduction

Although the association between instances of first human arrival and Quaternary faunal extinctions has long been recognized, significant debate persists regarding the strength of this correlation and the degree of human causation in extinction events (Martin, 1973, 1984; MacPhee and Marx, 1997; Martin and Steadman, 1999; Grayson and Meltzer, 2002, 2003; Barnosky et al., 2004; Lyons et al., 2004; Surovell et al., 2005, 2016; Koch and Barnosky, 2006; Surovell, 2007; Boulanger and Lyman, 2014; Stuart, 2015; Malhi et al., 2016; Broughton and Weitzel, 2018; Hixon et al., 2021; Prates and Perez, 2021; Lemoine et al., 2023; Svenning et al., 2024). Critical to the testing of hypotheses concerning anthropogenic or other causes of extinctions is the ability to accurately date both types of events, the initial archaeological appearance of humans and the last paleontological occurrence of extinct taxa. Herein I refer to faunal extinction dates as last appearance dates (LADs) and human colonization dates as first appearance dates (FADs). Several well-known issues complicate our ability to generate accurate LADs and FADs. Those include the temporal limit of radiocarbon dating (Webb, 1997; Jones, 1999; Miller et al., 1999; Price et al., 2011); sample contamination problems (Pike and Hedges, 2002; Herrando-Pérez, 2021; Herrando-Pérez and Stafford, 2025); redeposition and mixing of fossils, artifacts and sedimentary deposits (Waters, 1983; Price et al., 2011; Surovell et al., 2012, 2026); sample size issues and record incompleteness (Signor and Lipps, 1982; Roberts and Solow, 2003; Solow, 2005; Surovell et al., 2005; Faith and Surovell, 2009; Rivadeneira et al., 2009; Surovell and Grund, 2012; Wang and Marshall, 2016) and taphonomic bias (Surovell and Brantingham, 2007; Surovell et al., 2009; Surovell and Pelton, 2016; Bluhm and Surovell, 2019), or the tendency for younger things to be more common than older things in geologic contexts.

In paleontology, the Signor–Lipps effect refers to how differential preservation and variation in sample size can make a sudden catastrophic extinction event appear gradual in the fossil record (Signor and Lipps, 1982) because we are unlikely to find specimens dating very close to the extinction event in a sampled and incomplete geologic record. The reverse phenomenon, that the earliest example of a species known from the fossil record will post-date its origination, is known either as the Sppil–Rongis (Signor–Lipps backward) or Jaanusson effect (Jaanusson, 1976; Heads, 2012). Both problems pertain to human colonization (or other archaeological first appearance dates) and associated animal extinctions (Roberts and Solow, 2003; Solow, 2005; Rivadeneira et al., 2009; Wang and Marshall, 2016; Key et al., 2021). To state the obvious, it is so unlikely, as to verge on impossible, to identify the absolute last mammoth to have walked the North American continent, just as it is similarly unlikely to identify the first archaeological site created by humans in North America. While issues of extinction and origination of taxa are typically examined independently in paleontological contexts, in this study, I examine them together as the overkill hypothesis posits that human colonization and animal extinction are causally linked.

Here, I use a simple predator–prey simulation to examine expected age offsets associated with estimating the dates of human colonization and faunal extinction events. I define age offset as the difference between the true age of an event and the age inferred from the oldest or youngest sampled occurrence. For example, if human colonization occurred at 14,000 BP but the oldest known archaeological site dates to 13,400 BP, the estimated colonization age has an offset of 600 years. I argue that, under an overkill scenario, age offsets on last appearance dates (LADs) should be smaller than those on first appearance dates (FADs) given equal sample sizes. I then compare simulated results to empirical temporal frequency distributions of extinct fauna to evaluate whether real-world data exhibit the patterns predicted by the model. The results have important implications for understanding the timing of human colonization and faunal extinction worldwide.

## Methods

The simulation is designed to explore expected age offsets in LADs and FADs when sampling a human-driven extinction event. To be explicit, I am assuming extinction by overkill. The simulation could be modified to explore nonanthropogenic extinctions as well, but that is not my purpose. The simulation was written for R version 4.6.0 (R Core Team, 2026), and the code is provided in the Supplementary Information. The modeled scenario is designed to roughly approximate North American human colonization and mammalian extinction, but it could be easily modified to accommodate any time and place. It only includes the population dynamics of a single prey species. There are three parts to the simulation. First, I model human colonization and hunting of prey to extinction. Next, the resulting faunal and human population trends, which serve as proxies for archaeological and paleontological abundance, are modified to account for the age-dependent taphonomic loss of sites. Finally, I sample the archaeological and paleontological records to explore expected age offsets when estimating FADs and LADs.

In the base model, the simulation proceeds in annual increments and begins at 17,000 BP with an animal prey population that exists at carrying capacity (*K_a_*). Human and prey population dynamics were modeled using the discrete-time Verhulst, or logistic, growth equation. For humans, population size in year *t*+1 was calculated as:

(1) $$H_{t+1}=H_{t}+r_{h}H_{t}\left(1-\frac{H_{t}}{K_{h}}\right)$$

where *H_t_* is human population size in year *t*, *r_h_* is the maximum annual human population growth rate and *K_h_* is human carrying capacity. Prey population growth was modeled similarly, except that annual hunting mortality was subtracted after logistic growth:

(2) $$A_{t+1}=A_{t}+r_{a}A_{t}\left(1-\frac{A_{t}}{K_{a}}\right)-hH_{t}$$

where *A_t_* is prey population size at time *t*, *r_a_* is the maximum annual prey population growth rate, *K_a_* is prey carrying capacity and *h* is the per capita annual hunting rate.

In the base model, I set the prey carrying capacity (*K_a_*) to 100,000 individuals and *r_a_* is set to 0.14. Based on the regression reported by Caughley and Krebs (1983), this is the expected growth rate for an herbivorous mammal weighing ~700 kg, or roughly the size of the extinct North American Yesterday’s camel, *Camelops hesternus* (Smith et al., 2003). I use a founding human population (*H_0_*) of 100 individuals and set human carrying capacity (*K_h_*) to 10,000. I use a maximum annual human population growth rate (*r_h_*) of 0.01, or 1% per year based on observed typical rates of growth for recent foraging populations (Van Arsdale, 1978; Early and Headland, 1998; Gurven and Kaplan, 2007; Blurton Jones, 2016). Human population dynamics are independent of prey; I assume that with reduced numbers of prey, humans switch to alternative food resources. I set the *per capita* hunting rate (*h*) to 0.75. This is the number of prey animals taken per person per year. For each year, the simulation saves the number of people and prey in each population, and those numbers serve as proxies for initial archaeological and paleontological abundance prior to taphonomic effects.

To model the taphonomic loss of the fossil and archaeological records, I use empirically derived functions that describe the relationship between age and abundance of geologic deposits and paleontological occurrences. Taphonomic loss of the archaeological record is modeled using Equation 3 from Bluhm and Surovell (2019):

(3) $$n_{t}=8589.83{\left(t+1020.62\right)}^{-1.198}$$

a curve built from more than 4,000 global radiocarbon dates from noncultural terrestrial geologic deposits that describes the temporal frequency of dated sedimentary contexts over the last 40,000 years, where *n_t_* is the expected relative frequency of geologic contexts dating to time *t.* To modify the fossil record, I use the curve built from paleontological occurrences of extinct Pleistocene fauna for the contiguous United States from Surovell and Pelton (2016):

(4) $$n_{t}=2.055x10^{60}{\left(t+2914\right)}^{-14.05}$$

where *n_t_* is the expected relative frequency of fossil remains dating to time *t.* Taphonomic modification is accomplished by dividing human and animal population sizes by their respective taphonomic curves and then standardizing each series to its maximum value, so they range from 0 to 1. The resulting curves represent the expected relative abundance of extinct taxa and archaeological sites in the fossil and archaeological records.

I randomly sample each record and calculate extinction age offsets as the difference between the extinction date and the youngest sampled prey animal. Colonization age offset is the simulated colonization date (13,500 BP) minus the oldest sampled archaeological site.

To evaluate the sensitivity of model outcomes to parameter values, I conducted a global sensitivity analysis (Supplementary Information). Parameter values for founding human population size (*H_0_*), human population growth rate (*r_h_*), human carrying capacity (*K_h_*), prey population growth rate (*r_a_*), prey carrying capacity (*K_a_*) and per-capita hunting rate (*h*) are randomly sampled (*n* = 10,000) across predefined ranges (Supplementary Table S1). For each parameter combination, archaeological and paleontological records were simulated and sampled 30 times to estimate median FADs, LADs, age offsets and overlap rates.

I begin by examining the results of the global sensitivity analysis to evaluate the robustness of model outcomes across a broad range of parameter values. I then present the base model and examine expected age offsets for FADs and LADs under a representative overkill scenario. Additional experiments explore how archaeological and paleontological sample sizes affect age offsets and how variation in human population growth rates influences time to extinction, FADs, LADs and colonization and extinction age offsets.

To compare simulated to actual data, I compiled six examples of temporal frequency distributions of extinct fauna that differ dramatically in age and geography (Supplementary Data 1). Those include amino acid racemization dates on *Genyornis* eggshell (*n* = 226) from the Lake Eyre region, Australia (Miller et al., 2005), radiocarbon dates (*n* = 90) of North American Mastodon (Widga et al., 2017), radiocarbon dates (*n* = 132) of Eurasian *Megaloceros* (Lister and Stuart, 2019), radiocarbon dates (*n* = 268) of extinct South American megafauna (Prates and Perez, 2021), radiocarbon dates (*n* = 215) of extinct fauna in Madagascar (Hixon et al., 2021) and radiocarbon dated moas (*n* = 644) from New Zealand (Perry et al., 2014). For each dataset, I removed problematic dates, including infinite and modern radiocarbon ages, or samples with missing error terms. I calibrated radiocarbon ages using the appropriate terrestrial calibration curve, either IntCal20 (Reimer et al., 2020) or SHCal20 (Hogg et al., 2020) and Bchron v. 4.7.7 for R (Haslett and Parnell, 2008). For the five radiocarbon datasets, I calculated summed probability distributions for comparison to simulated distributions.

## Results

**Figure 1.**
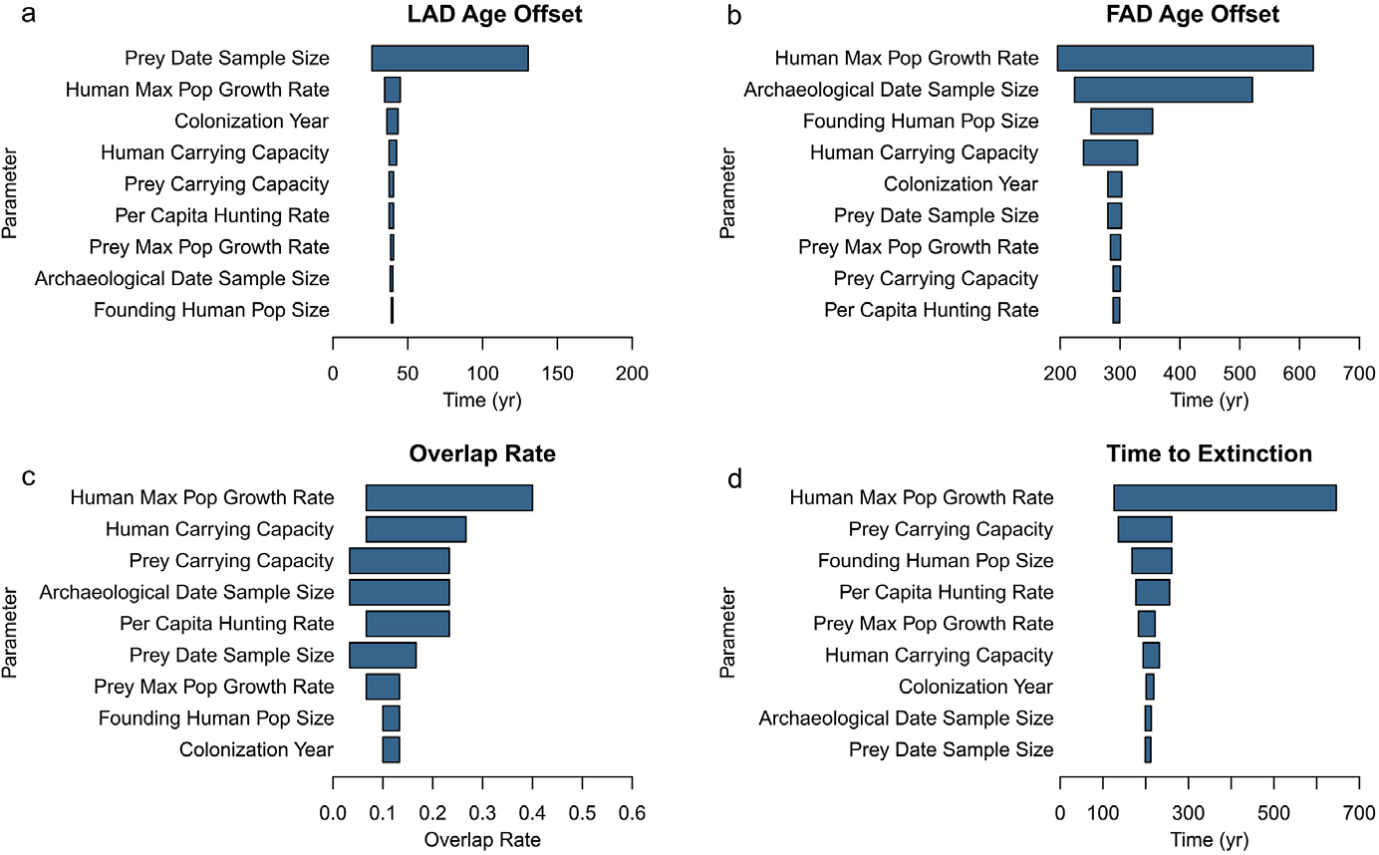
Tornado plots illustrating the sensitivity of model outcomes to variation in input parameters. Bars represent the range in the median value of each output variable across all tested values of a given input parameter while averaging over variation in all other parameters. Longer bars indicate greater influence of the parameter on model outcomes. Results are shown for (a) median LAD age offset, (b) median FAD age offset, (c) overlap rate and (d) time to extinction. Figure 1. long description.

Results of the sensitivity analysis demonstrate that the central conclusion of the model is robust across a broad range of parameter values (Supplementary Information). Of 10,000 randomly selected parameter combinations, 9,910 (99.1%) produced prey extinction. Nonextinction cases were characterized by low human carrying capacities (*K_h_*) and per capita hunting rates (*h*), combined with high prey carrying capacities (*K_a_*) and population growth rates (*r_a_*), indicating that prey populations could replace losses to hunting and persist throughout the simulation. Also, across extinction simulations, LAD age offsets were consistently smaller than FAD age offsets. LAD age offsets were influenced primarily by the sample size of dated prey remains (Figure 1a), whereas FAD age offsets were strongly affected by human population growth rate and archaeological sample size (Figure 1b). Founding human population size (*H_0_*) and carrying capacity (*K_h_*) had comparatively little effect on FAD age offsets. Human population growth rate also emerged as the strongest predictor of overlap rates and time to extinction (Figure 1c,d). Because sample size and human population growth rate exerted the greatest influence on model outcomes, I focus on these variables in the experiments presented later. I begin with a representative base model and then examine how variation in sample size and human population growth rate affects age offsets, overlap rates and extinction timing.

**Figure 2.**
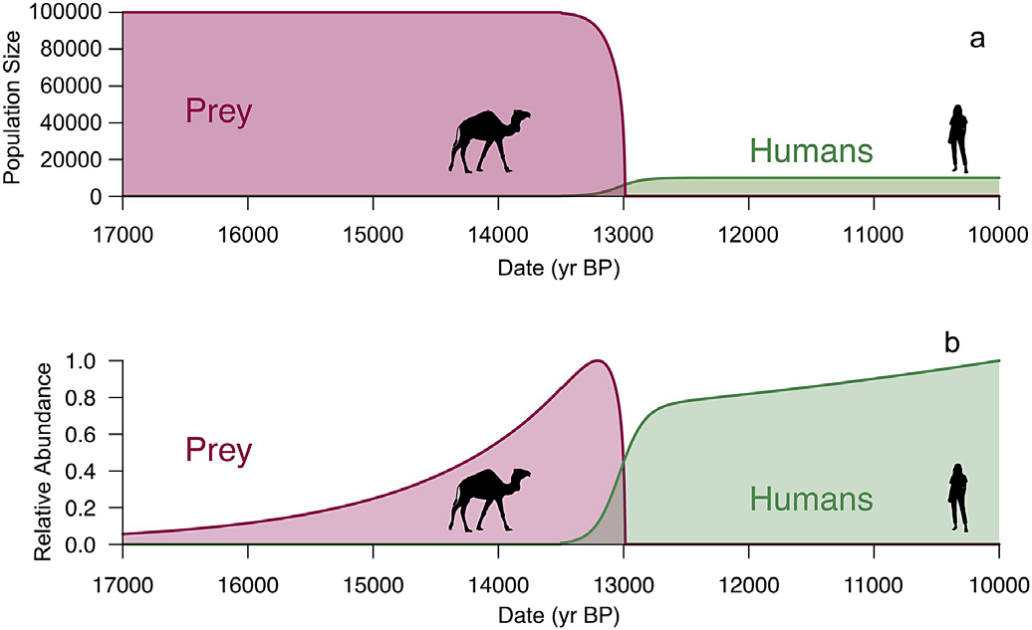
Simulated population sizes of humans and prey (a) and the relative abundance of each in the archaeological and paleontological records after accounting for taphonomic bias and standardizing each record to its respective maximum value (b). Thus, panel b illustrates temporal changes in relative abundance and should not be interpreted as showing differences in the absolute quantities of archaeological and paleontological materials. The scenario shown is the base model (*K_a_* = 100,000; *K_h_* = 10,000; *r_a_* = 0.14; *r_h_* = 0.01; *H_0_* = 100). Figure 2. long description.

In the base model of the simulation, prey populations initially decline gradually with human colonization, but as human populations grow, prey numbers drop precipitously (Figure 2a). Extinction is complete in 12,986 BP, or 514 years after the human founding event. At a modest 1% per year rate of growth, human populations reach 90% of carrying capacity after 681 years and 99% after 920 years. After taphonomic modification of the record, fossil occurrences of prey gradually increase over time, reaching a peak at 13,208 BP, almost 300 years after human colonization, and then drop quickly. Abundances of archaeological materials superficially track human population size, although the older parts of the record are reduced relative to more recent dates (Figure 2b). This is especially apparent in the portion of the curve once carrying capacity has been reached and population remains constant, but because of taphonomic bias, human population appears to grow gradually.

**Figure 3.**
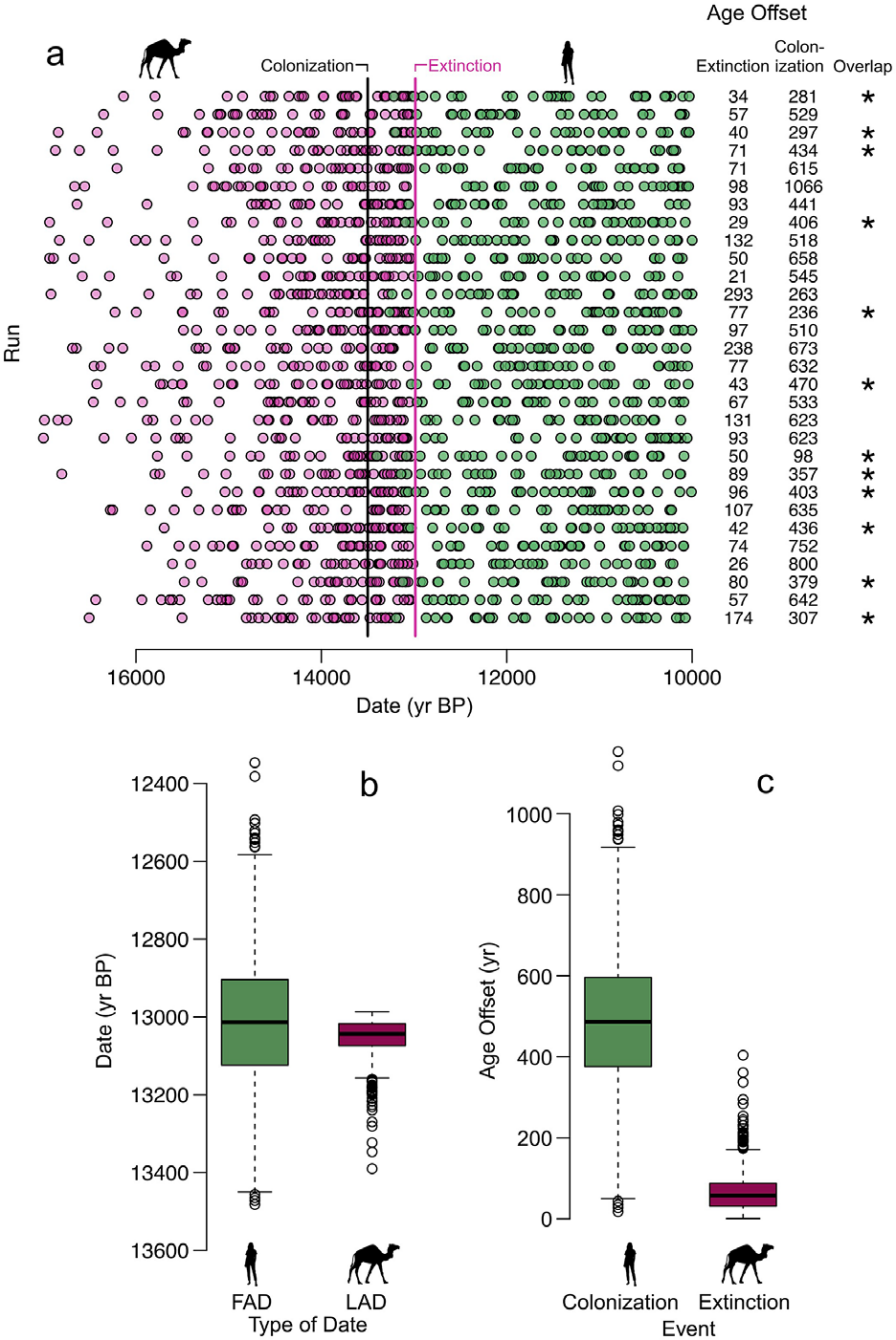
Results of sampling the simulated archaeological and fossil records from the base model. (a) Simulated radiocarbon ages from sampling the fossil and archaeological records (*n* = 30 each). Each horizontal line represents a different run of the simulation. Extinction and colonization age offsets for each run are shown to the right in addition to whether the oldest date on archaeological sites overlaps in time with the youngest date on extinct fauna. Cases where overlap occurs are marked with a star (*). (b) Boxplots of FADs and LADs for 1,000 runs of the simulation. (c) Boxplots of colonization and extinction age offsets for 1,000 runs of the simulation. Figure 3. long description.

In Figure 3a, I show the results of equally sampling the archaeological and fossil records for 30 runs of the simulation. For each run, I randomly generated 30 dates from the taphonomically adjusted archaeological and fossil records. Under the simulated conditions, finding faunal specimens relatively close to the extinction date is a common occurrence, but the earliest archaeological sites typically occur 400 to 600 years after colonization. Furthermore, with these sample sizes, temporal overlap between the oldest archaeological and youngest paleontological dates only occurs in about 43% of cases. This means that in more than half of the cases under these conditions, it would appear that the prey suffered extinction prior to human arrival, when in fact, they coexisted for more than five centuries.

Under the conditions of the base model, with sample sizes of 30, median FADs and LADs are similar. For 1,000 runs of the simulation, the median FAD for archaeological sites is 13,014 BP, and the median LAD for extinct fauna is slightly earlier at 13,044 BP (Figure 3b). Again, under these conditions, the fossil and archaeological records would on average suggest that animals went extinct prior to human arrival. While FADs and LADs for both sets of dates are similar, age offsets are very different (Figure 3c). The median age offset when estimating the date of extinction is only 58 years, while the median age offset for estimating colonization is 486 years, or almost an order of magnitude higher. This difference arises from the shapes of the temporal frequency distributions for archaeological sites and extinct animals. Due to the dual effects of taphonomic bias and hunting-driven rapid population decline, occurrences of extinct fauna are expected to be most common close to the extinction event (Figure 2b). By contrast, archaeological sites are expected to be rarest when human population levels are lowest, at or near the colonization event. Taphonomic bias exacerbates that rarity. Therefore, in an overkill scenario with equal sampling of the fossil and archaeological records, age offsets for extinction should be much smaller than those for colonization.

**Figure 4.**
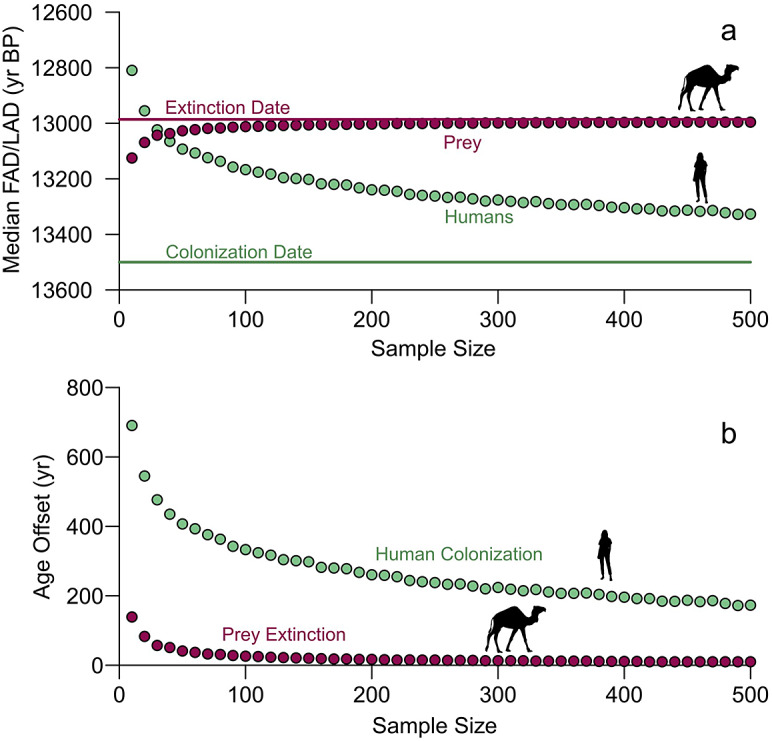
The effects of modifying sample size on FADs and LADs (a) and age offsets (b). Figure 4. long description.

As sample sizes are increased, FADs and LADs asymptotically approach colonization and extinction dates, their theoretical limits (Figure 4a). At sample sizes of 500, age offsets on extinction are reduced to fewer than 10 years, while age offsets on colonization remain high with a median value of 179 years (Figure 4b). In fact, the relative magnitude of these age offset differences increases. Again, this result reinforces the idea that finding fauna dating close to extinction should be easier than finding archaeological sites close to the colonization date, regardless of sample size. That said, even at modest rates of human population growth and small sample sizes, finding archaeological sites within the first millennium following human arrival should be possible.

**Figure 5.**
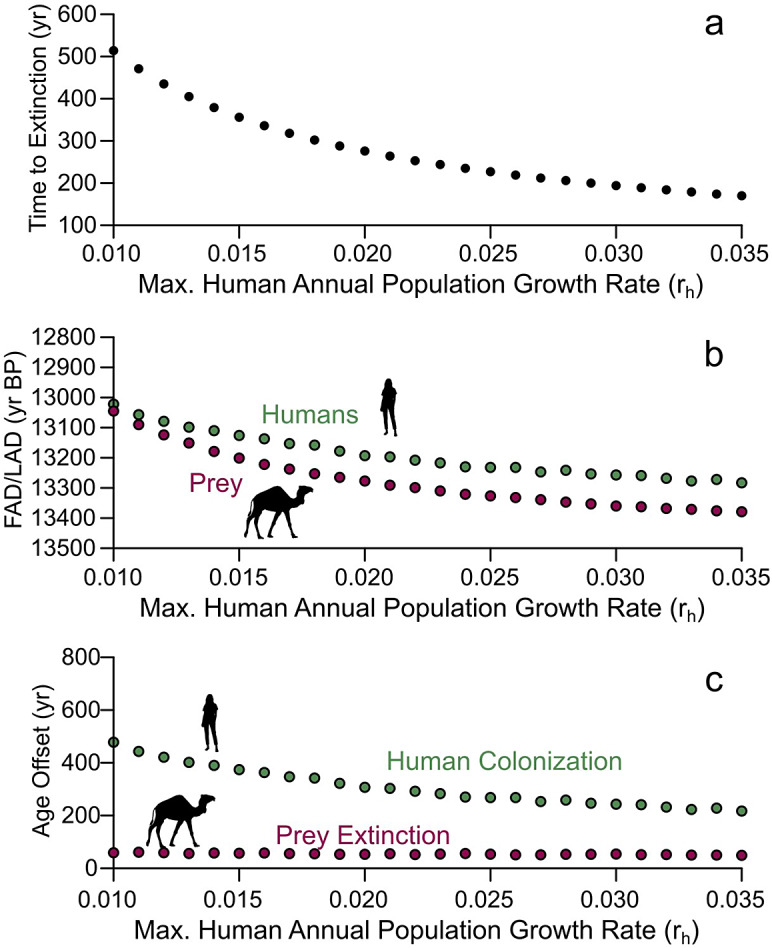
The effects of modifying maximum human population growth rate (r_h_) on time to extinction (a), FADs and LADs (b) and age offsets (c). Figure 5. long description.

As human population growth rate (r_h_) is increased from 0.01 to 0.035, the model behaves in predictable ways. The time to extinction in the base model is reduced from 514 to 170 years as human populations quickly reach carrying capacity (Figure 5a). Expected FADs and LADs both get older (Figure 5b). At a growth rate of 3.5% per year, the oldest archaeological site found should date to a median age of ca. 13,277 BP, or an age offset of only 223 years. As animal extinction occurs more rapidly when human populations grow quickly, the youngest faunal specimens sampled are also pushed back in time to 13,377 BP. Although age offsets of FADs decline by about 254 years when increasing r_h_ from 0.01 to 0.035, age offsets on LADs are only marginally affected, dropping from 59 to 47 years (Figure 5c). Importantly, varying the rate of human population growth does not change the relative difficulty of dating extinction and colonization. Extinction age offsets are consistently expected to be smaller than those on colonization.

**Figure 6.**
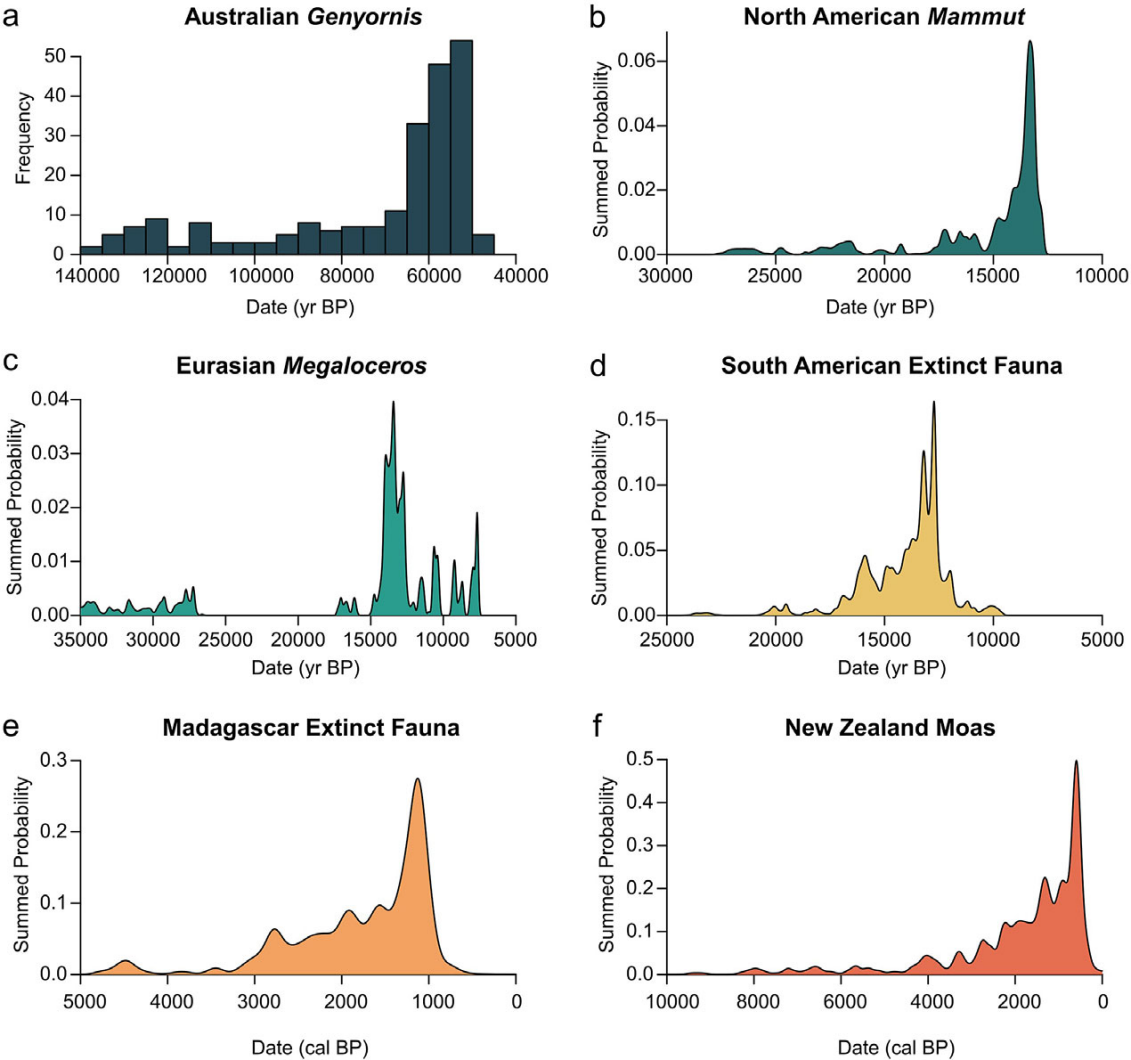
Six examples of temporal frequency distributions of directly dated extinct Quaternary faunas from islands and continents. (a) Histogram of mean amino acid racemization dates on *Genyornis* eggshell from Lake Eyre, Australia. (b) Summed probability distribution of calibrated radiocarbon ages of North American mastodon. (c) Summed probability distribution of calibrated radiocarbon ages of Eurasian *Megaloceros.* (d) Summed probability distribution of calibrated radiocarbon ages of extinct South American taxa. (e) Summed probability distribution of calibrated radiocarbon ages of extinct fauna from Madagascar. (f) Summed probability distribution of calibrated radiocarbon ages of New Zealand moas. Figure 6. long description.

Figure 6 shows frequency distributions of dates on extinct fauna from around the world. These examples vary in age span from 140,000 to 45,000 (Australia) to 10,000 to 0 BP (New Zealand). As predicted by the simulation, each case shows a strongly left-skewed distribution (with time advancing to the right on the x-axis) with a strong peak preceding extinction. This pattern holds regardless of age and geography and characterizes dates of extinct fauna from continents and islands. These distributions, however, do differ from the simulations in one important way. In every case, there is a right tail to the distribution. In most cases, the right tail is short, as in Australia (Figure 6a), North America (Figure 6b), Madagascar (Figure 6e) and New Zealand (Figure 6f). In two cases, the tail extends well beyond the mode. In South America, there are dated faunal specimens more than 2,000 years younger than the modal probability (Figure 6d) and in Eurasia, *Megaloceros* appears to persist more than 5,000 years after the distribution peaks from 14,000 to 13,000 BP. The presence of right tails in every case is likely in part a result of dating errors, such as those caused by radiocarbon dating of bone collagen, where samples tend to yield ages that are artificially young when affected by small amounts of modern or younger contamination (Hedges and Law, 1989; Taylor, 1992; Higham et al., 2006; Devièse et al., 2018; Herrando-Pérez, 2021). By adding an error term to sampling in the simulation, in most cases, these tails could be replicated. While the accuracy of Holocene dates on South American extinct fauna has been questioned (Politis et al., 2019; Prates and Perez, 2021), the case of *Megaloceros* in Eurasia (Lister and Stuart, 2019), however, may truly show long-term survival of an extinct taxon after a major population reduction in the Late Pleistocene, perhaps due to a time- and space-transgressive extinction event as human populations expanded into northern and montane regions in the Holocene. My simulation, it should be noted, has no spatial component, which might explain this difference. Alternatively, this case might represent a nonanthropogenic extinction event.

## Discussion

An a priori assumption about dating extinction and colonization might be that age offsets of FADs and LADs should be somewhat symmetrical, as both involve the chance discovery of something near to the theoretical limits of their distributions. Significant age offsets are expected of small growing human populations after a founding event because people are thinly spread and growth rates are limited by population size and human biology. Large age offsets are not necessarily expected in the case of extinction by overkill. There are few limits to the rates at which population numbers can decline, and in the case of the introduction of a new predator to an ecosystem, sudden population collapse might be expected. Thus, one important implication of these simulations is that it should not be difficult to find examples of extinct fauna dating close to the time of extirpation in the Quaternary when extinction was sudden and catastrophic. Again, for the purposes of this paper, I assume extinction by overkill. In the case of a prolonged extinction, one in which a population declines over thousands of years whether caused by human hunting or some other factor, this may not be true.

Even though age offsets for human FADs are expected to be greater than those for faunal LADs, under modest rates of population growth and relatively small sample sizes, the offsets are not absolutely large. For example, with a sample size of only 30 dates from the first 3,500 years of human occupation following a 13,500 BP colonization event, it should not be difficult to find at least one site dating from the first millennium of human presence. This is because humans are expected to reach carrying capacity fairly quickly, even at modest population growth rates. If human populations grow quickly after colonization, it should not be difficult to find sites within the first few hundred years of human presence. These factors likely explain why archaeologists were able to push back colonization dates to significant antiquity in the early history of archaeological investigation on multiple continents (e.g., Kelly 2003). That human populations should quickly approach carrying capacity, determined by ecological productivity and subsistence economy, means that the period during which it will be challenging to find evidence of a human presence should be brief in archaeological terms, probably measured in centuries, not millennia, which highlights problems in need of explanation when anomalously early sites are proposed (e.g., Clarkson et al., 2017; Hansford et al., 2018; Davis et al., 2019; Bennett et al., 2021). There are few realistic demographic scenarios that would allow human populations to remain archaeologically cryptic for thousands of years after initial appearance.

There is one additional factor that might reduce age offsets on colonization (FADs). Humans make artifacts; animals do not. While paleontologists are largely dependent on finding fossil remains of animals themselves, except in rare cases that preserve ichnofossils (e.g., Martin et al., 1961; Mead et al., 1986; Hunt and Lucas, 2018), archaeologists can identify human presence from a wide variety of material traces. In effect, humans amplify their signature in the geologic record by producing archaeological materials throughout the landscapes they occupy. Although this process is difficult to model explicitly, an individual forager may create hundreds of archaeological deposits during a lifetime. This amplification could substantially increase the number of observable archaeological occurrences and thereby reduce age offsets associated with colonization. Importantly, while artifact production increases the absolute abundance of archaeological materials, it should not fundamentally alter their temporal distribution. As a result, the principal conclusions of this modeling exercise are likely robust. Indeed, if archaeological materials are substantially more abundant than fossil remains, it may be easier to identify 30 early archaeological sites than 30 dateable specimens of an extinct animal species, further increasing the contrast between the precision of colonization and extinction estimates.

As age offsets on LADs for extinct fauna should be relatively small, even with small samples of high-quality radiocarbon dates, the debate concerning sudden catastrophic mass extinction following human arrival vs. slow staggered extinctions across a range of taxa (Grayson and Meltzer, 2002; Faith and Surovell, 2009; Meltzer, 2015, 2020) should be resolvable. Additional work attempting to date underrepresented Quaternary taxa could be very worthwhile, notwithstanding the challenges mentioned earlier in obtaining accurate dates for any given fossil specimen.

Finally, my simulations assume random sampling of the fossil and archaeological records. While large samples of dates produced by many researchers working on many different problems across many regions might produce samples that approach representativeness, there are reasons to expect that in reality sample biases may come into play. For example, some well-known fossil localities from which many dates have been produced (e.g., Trueman et al., 2005; Seersholm et al., 2020; O’Keefe et al., 2023; Redman et al., 2023) do not span the entirety of the period of interest. Furthermore, examples of fauna dating close to extinction and archaeological sites close to colonization in time are likely to receive disproportionate research attention and dating. Such biases might affect temporal frequency distributions like those shown in Figure 6. Nonetheless, the overall distribution of dates on extinct fauna in many parts of the world are similar to those predicted by the simulations presented here.

## Conclusions

Using a simple predator–prey simulation that assumes an overkill scenario, I have shown that age offsets on LADs for extinct fauna should be consistently smaller than those for FADs of human colonization at equal sample sizes. This asymmetry arises because the modeled rapid, hunting-driven population collapse of prey causes a peak in fossil occurrences close to the extinction event, whereas the initial rarity of small, growing human populations, exacerbated by taphonomic bias, makes finding the earliest archaeological sites more challenging. This conclusion is also insensitive to variation in human population growth rates. The simulations reinforce the theoretical expectation that a sudden, catastrophic Quaternary extinction should be readily resolvable from the fossil record, a pattern consistent with real-world temporal frequency distributions of extinct megafauna, although they require sufficient sample size to reduce age offsets. With small sample sizes of dated fauna and/or archaeological sites, a false appearance of extinction prior to human arrival is expected. These results also have important implications for understanding the timing of human colonization events, showing the period during which human presence could remain archaeologically cryptic is likely brief, measured in centuries rather than millennia.

## Supporting information

10.1017/ext.2026.10017.sm001 Surovell supplementary material 1 Surovell supplementary material

10.1017/ext.2026.10017.sm002 Surovell supplementary material 2 Surovell supplementary material

## Data Availability

No novel empirical datasets were generated during this study. All empirical data used in the analyses were obtained from previously published sources cited in the References. Those dates are also included in the supplementary information. The simulation code and output data supporting the findings of this study are available from the corresponding author upon reasonable request.

## Supplementary material

The supplementary material for this article can be found at http://doi.org/10.1017/ext.2026.10017.

## Long descriptions

### Figure 1. Long description

Four horizontal tornado plots rank the influence of nine model parameters on four model outputs. Panel (a) shows that LAD age offset is overwhelmingly controlled by prey date sample size, while all other parameters have much smaller effects. Panel (b) shows that FAD age offset is most strongly affected by maximum human population growth rate, followed by archaeological date sample size, with founding human population size and human carrying capacity having moderate effects. Panel (c) shows that overlap rate is influenced primarily by human population growth rate, with human and prey carrying capacities, archaeological sample size, and hunting rate also contributing. Panel (d) shows that time to extinction is dominated by human population growth rate, whereas all remaining parameters have comparatively minor effects. Overall, the sensitivity analysis demonstrates that extinction age estimates depend mainly on paleontological sampling, whereas colonization estimates depend primarily on human demography and archaeological sampling.

Navigate back to Figure 1..

### Figure 2. Long description

Panel (a) plots population size through time. Prey begin at carrying capacity and remain stable until human colonization at 13,500 years BP. As the human population grows, prey decline gradually at first and then collapse rapidly to extinction at approximately 12,986 BP. Humans continue increasing until approaching carrying capacity after prey extinction. Panel (b) shows the expected archaeological and paleontological records after applying taphonomic bias and standardizing both records. Relative abundance of prey remains increases toward the extinction event and then falls abruptly to zero. Archaeological abundance is initially very low after colonization because human populations are small, then rises steadily as the population grows and because younger deposits are better preserved. The figure illustrates why fossils are expected to cluster near extinction while the earliest archaeological evidence remains comparatively sparse.

Navigate back to Figure 2..

### Figure 3. Long description

Panel (a) displays 30 independent sampling runs. Purple points represent sampled prey fossils and green points represent archaeological dates. Vertical lines mark the true colonization and extinction dates. In most runs, the youngest fossil occurs close to extinction, whereas the oldest archaeological site occurs several centuries after colonization. Asterisks identify the relatively few runs in which archaeological and paleontological dates overlap in time. Panel (b) shows boxplots of estimated first appearance dates (FADs) and last appearance dates (LADs) from 1,000 simulations. Median FADs and LADs are similar even though their true events differ substantially. Panel (c) shows boxplots of age offsets, demonstrating that extinction estimates have much smaller errors than colonization estimates. The figure illustrates that limited sampling can create the false appearance that extinction preceded human arrival even when humans and prey coexisted.

Navigate back to Figure 3..

### Figure 4. Long description

Panel (a) shows median estimated FADs and LADs as sample size increases from 10 to 500 observations. Both estimates converge toward their true values as additional dates are collected, but extinction dates converge much more rapidly than colonization dates. Horizontal reference lines indicate the true extinction and colonization dates. Panel (b) plots age offset against sample size. Colonization age offset declines gradually from nearly 700 years to approximately 180 years, whereas extinction age offset falls rapidly from roughly 140 years to fewer than 10 years. Increasing sample size therefore improves both estimates but has a much greater effect on the precision of extinction dates.

Navigate back to Figure 4..

### Figure 5. Long description

Panel (a) shows that increasing the maximum annual human population growth rate shortens the time from colonization to extinction, reducing it from more than 500 years to less than 200 years. Panel (b) shows that higher growth rates shift both estimated human colonization dates and prey extinction dates closer to the true colonization event because prey are driven extinct more rapidly. Panel (c) shows corresponding age offsets. Colonization age offset decreases substantially as growth rate increases, whereas extinction age offset changes only slightly and remains consistently small. The figure demonstrates that faster human population growth improves estimates of colonization timing but does not alter the fundamental result that extinction dates are estimated more accurately than colonization dates.

Navigate back to Figure 5..

### Figure 6. Long description

Six panels present dated records of extinct fauna from Australia, North America, Eurasia, South America, Madagascar and New Zealand. Australia is shown as a histogram of amino acid racemization dates, whereas the remaining panels show summed probability distributions of calibrated radiocarbon ages. Despite large differences in geography, age and taxonomic composition, all six datasets exhibit a similar left-skewed distribution (with time advancing to the right) in which dated specimens become increasingly common toward the extinction interval before declining abruptly. Most datasets have only a short tail of younger dates after the peak, although Eurasian Megaloceros and South American megafauna display longer tails that may reflect prolonged survival, dating uncertainty or spatial variation in extinction timing. The figure shows that empirical fossil records closely resemble the temporal pattern predicted by the simulation.

Navigate back to Figure 6..
