# Supplementary material for: Why it is easier to date Quaternary extinctions than human colonizations under conditions of overkill
Source: Camb Prism Extinct. 2026 Jul 21;4:e9. doi: 10.1017/ext.2026.10017 (PMC13430543; doi:10.1017/ext.2026.10017)
Supplement: Surovell supplementary material 2 — Surovell supplementary material [file S2755095826100175sup002.docx]

**Sensitivity Analysis**

To identify the parameters driving variation in model outputs (LAD age offset, FAD age offset, overlap rate, and time to extinction), I conducted a sensitivity analysis. I constructed a factorial parameter space containing all combinations of input parameter values (Table S1), resulting in 1.584 × 10^8^ possible parameter combinations. From this set, I randomly sampled 10,000 parameter combinations without replacement for analysis. For these simulations, colonization year was also varied from 22,000 to 13,000 BP, and the start and end dates of each simulation were set to 3,500 years before and after the colonization year, respectively. Because no LAD or extinction age offset can be defined when extinction does not occur, simulations in which prey remained extant (n = 90; 0.9%) were excluded from sensitivity analyses. For the remaining simulations, I generated tornado plots showing the range of variation in the median value of each output variable across levels of each input parameter (Figure 1). I also fit multiple linear regression models to quantify the partial contribution of each input parameter to variation in model outcomes. Because the four dependent variables were measured on different scales, each was standardized to a mean of zero and a standard deviation of one prior to regression analysis.

*Table S1. Input parameter ranges and increments for sensitivity analysis*

| **Input Parameter** | **Min. Value** | **Max. Value** | **Increment** |
| --- | --- | --- | --- |
| Founding Human Pop. Size (*H_0_*) | 100 | 500 | 100 |
| Max. Human Annual Pop. Growth Rate (r*_h_*) | 0.005 | 0.03 | 0.0025 |
| Human Carrying Capacity (K*_h_*) | 10,000 | 40,000 | 2,000 |
| Max. Prey Annual Pop. Growth Rate (r*_a_*) | 0.1 | 0.2 | 0.02 |
| Prey Carrying Capacity (*K_a_*) | 40,000 | 200,000 | 40,000 |
| Per Capita Hunting Rate (*h*) | 0.6 | 1.6 | 0.2 |
| Colonization Year in yr BP | 13,000 | 22,000 | 1,000 |
| Prey ^14^C Sample Size (*n_a_*) | 10 | 100 | 10 |
| Archaeological ^14^C Sample Size (*n_h_*) | 10 | 100 | 10 |

**LAD Age Offset**

LAD age offset, defined as the difference between the prey extinction date and the youngest radiocarbon date obtained from a fossil specimen, was the least sensitive of the four output variables. It was influenced primarily by the sample size of radiocarbon dates on prey remains (*n_a_*) (Figure 1a). Increasing prey date sample size from 10 to 100 reduced median LAD age offset from approximately 131 to 26 years. The remaining eight input variables had comparatively little effect. Only one other parameter altered median LAD age offset by more than 10 years across its tested ranges, maximum human population growth rate (*r_h_*), which changed median LAD age offset by 10.5 years.

*Table S2. LAD median age offset in the sensitivity analysis expressed as median minimum, maximum, and range, after aggregation by parameter value.*

| **Input Parameter** | **Min (yr)** | **Max (yr)** | **Range (yr)** |
| --- | --- | --- | --- |
| Prey Date Sample Size (*n_a_*) | 26.0 | 130.5 | 104.5 |
| Max. Human Annual Pop. Growth Rate (r*_h_*) | 34.5 | 45.0 | 10.5 |
| Colonization Year in yr BP | 36.0 | 43.5 | 7.5 |
| Human Carrying Capacity (*K_h_*) | 37.5 | 42.5 | 5.0 |
| Prey Carrying Capacity (*K_a_*) | 37.5 | 40.5 | 3.0 |
| Per Capita Hunting Rate (*h*) | 37.5 | 40.5 | 3.0 |
| Max. Prey Annual Pop. Growth Rate (r*_a_*) | 38.5 | 40.5 | 2.0 |
| Archaeological ^14^C Sample Size (*n_h_*) | 38.3 | 40.0 | 1.8 |
| Founding Human Pop Size (*H_0_*) | 39.0 | 40.0 | 1.0 |

*Table S3. Results of a multiple linear regression analysis examining the input parameters on standardized median LAD age offset. The table reports unstandardized regression coefficients (Estimate), standard errors, t-statistics, p-values, significance levels, and partial coefficients of determination (partial R²).*

| **Variable** | **Estimate** | **Std Error** | **t** | **Partial r^2^** | **p** |
| --- | --- | --- | --- | --- | --- |
| Founding Human Pop. Size (*H_0_*) | 0.000 | 0.001 | -0.23 | 0.000 | 0.82 |
| **Max. Human Annual Pop. Growth Rate (r*_h_*)** | **-499.254** | **26.819** | **-18.62** | **0.034** | **<<0.001^***^** |
| **Human Carrying Capacity (*K_h_*)** | **0.000** | **0.000** | **-5.71** | **0.003** | **<<0.001^***^** |
| **Max. Prey Annual Pop. Growth Rate (r*_a_*)** | **-17.411** | **6.238** | **-2.79** | **0.001** | **0.005^**^** |
| **Prey Carrying Capacity (*K_a_*)** | **0.000** | **0.000** | **6.56** | **0.004** | **<<0.001^***^** |
| **Per Capita Hunting Rate (*h*)** | **-3.268** | **0.620** | **-5.27** | **0.003** | **<<0.001^***^** |
| **Colonization Year in yr BP** | **0.001** | **0.000** | **20.04** | **0.039** | **<<0.001^***^** |
| **Prey ^14^C Sample Size (*n_a_*)** | **-0.931** | **0.007** | **-126.22** | **0.617** | **<<0.001^***^** |
| Archaeological ^14^C Sample Size (*n_h_*) | 0.000 | 0.007 | -0.07 | 0.000 | 0.948 |
| *Note: Lines in bold face denote a significant result. Significance levels are denoted as follows: * p < 0.05; ** p < 0.01; *** p < 0.001.* | | | | | |

**FAD Age Offset**

FAD age offset, defined as the difference between the human colonization date and the oldest sampled archaeological site after taphonomic correction, was strongly influenced by human demographic variables and archaeological sample size (Figure 1b, Tables S4 and S5). Among the demographic parameters, maximum human population growth rate (*r_h_*) had the largest effect. Increasing growth rate from 0.5% to 3% per year reduced median FAD age offset from 623 to 196 years. Archaeological sample size had a similarly large influence, reducing FAD age offset from 521 to 224 years across the range of values examined. Founding population size (*H_0_*) and human carrying capacity (*K_h_*) had more modest effects, altering median FAD age offset by approximately 103 and 90 years, respectively. Multiple regression results were consistent with the tornado plots, identifying human population growth rate and archaeological sample size as the strongest predictors of FAD age offset, with partial r² values of 0.66 and 0.499, respectively (Table S5). Faster population growth and larger archaeological samples both increase the probability of detecting archaeological evidence close to the true colonization date, thereby reducing FAD age offset.

Importantly, FAD age offset was largely insensitive to the timing of human colonization. Across all colonization dates examined, median FAD age offset varied by only about 24 years and remained within a relatively narrow range of approximately 280–303 years. Thus, even when archaeological sample sizes are small, the expected discrepancy between the true colonization date and the oldest dated archaeological site is on the order of centuries rather than millennia. This result suggests that very large gaps between estimated colonization dates and the ages of the oldest archaeological sites are unlikely to arise from sampling effects alone.

*Table S4. FAD median age offset in the sensitivity analysis expressed as median minimum, maximum, and range, after aggregation by parameter value.*

| **Input Parameter** | **Min (yr)** | **Max (yr)** | **Range (yr)** |
| --- | --- | --- | --- |
| Max. Human Annual Pop. Growth Rate (r*_h_*) | 195.5 | 623.0 | 427.5 |
| Archaeological ^14^C Sample Size (*n_h_*) | 224.0 | 521.3 | 297.3 |
| Founding Human Pop. Size (*H_0_*) | 251.5 | 354.5 | 103.0 |
| Human Carrying Capacity (*K_h_*) | 239.0 | 329.3 | 90.3 |
| Colonization Year in yr BP | 279.5 | 303.0 | 23.5 |
| Prey ^14^C Sample Size (*n_a_*) | 279.5 | 302.5 | 23.0 |
| Max. Prey Annual Pop. Growth Rate (r*_a_*) | 284.0 | 301.0 | 17.0 |
| Prey Carrying Capacity (*K_a_*) | 288.5 | 300.5 | 12.0 |
| Per Capita Hunting Rate (*h)* | 288.5 | 299.5 | 11.0 |

*Table S5. Results of a multiple linear regression analysis examining the input parameters on standardized median FAD age offset. The table reports unstandardized regression coefficients (Estimate), standard errors, t-statistics, p-values, significance levels, and partial coefficients of determination (partial r²).*

| **Variable** | **Estimate** | **Std Error** | **t** | **Partial r^2^** | **p** |
| --- | --- | --- | --- | --- | --- |
| **Founding Human Pop. Size (*H_0_*)** | **-0.270** | **0.006** | **-45.75** | **0.175** | **<<0.001^***^** |
| **Max. Human Annual Pop. Growth Rate (r*_h_*)** | **-14667.949** | **105.934** | **-138.46** | **0.660** | **<<0.001^***^** |
| **Human Carrying Capacity (*K_h_*)** | **0.003** | **0.000** | **35.16** | **0.111** | **<<0.001^***^** |
| Max. Prey Annual Pop. Growth Rate (r*_a_*) | 15.348 | 24.641 | 0.62 | 0.000 | 0.533 |
| Prey Carrying Capacity (*K_a_*) | 0.000 | 0.000 | -0.17 | 0.000 | 0.866 |
| **Per Capita Hunting Rate (*h*)** | **-5.239** | **2.448** | **-2.14** | **0.001** | **0.032^*^** |
| **Colonization Year in yr BP** | **-0.001** | **0.000** | **-3.18** | **0.001** | **0.002^**^** |
| Prey ^14^C Sample Size (*n_a_*) | 0.039 | 0.029 | 1.35 | 0.000 | 0.176 |
| **Archaeological ^14^C Sample Size (*n_h_*)** | **-2.870** | **0.029** | **-99.19** | **0.499** | **<<0.001^***^** |
| *Note: Lines in bold face denote a significant result. Significance levels are denoted as follows: * p < 0.05; ** p < 0.01; *** p < 0.001.* | | | | | |

**Overlap Rate**

Unlike LAD and FAD age offsets, which were dominated by a small number of parameters, overlap rates were influenced by a broader range of human demographic, prey demographic, and sampling variables (Tables S6 and S7). Human population growth rate (*r_h_*) was the strongest predictor of overlap rate (partial r^2^=35.7%) in the multiple regression model. Increasing human population growth rates reduced overlap because rapidly growing human populations drove prey populations to extinction more quickly. In cases where human populations grow slowly, it should be easier to detect temporal overlap with smaller samples. In contrast, larger prey carrying capacities (*K_a_*) and higher prey population growth rates (r^a^) increased overlap by prolonging coexistence between humans and prey. Sampling intensity also had a substantial effect. Archaeological sample size (*n_h_*) and prey radiocarbon sample size (*n_a_*) were also important predictors (partial r^2^=0.276 and .086, respectively) of the variation in overlap rates indicating that overlap is influenced not only by ecological processes but also by the intensity of archaeological and paleontological sampling. Colonization year and founding human population size had little effect on overlap rates. Overall, these results suggest that the degree of apparent overlap between humans and prey is determined primarily by the balance between human population growth, prey population resilience, and the intensity with which both records are sampled.

*Table S6. Rate of overlap in the sensitivity analysis expressed as median minimum, maximum, and range, after aggregation by parameter value.*

| **Input Parameter** | **Min** | **Max** | **Range** |
| --- | --- | --- | --- |
| Max. Human Annual Pop. Growth Rate (r*_h_*) | 0.067 | 0.400 | 0.333 |
| Human Carrying Capacity (K*_h_*) | 0.067 | 0.267 | 0.200 |
| Prey Carrying Capacity (*K_a_*) | 0.033 | 0.233 | 0.200 |
| Archaeological ^14^C Sample Size (*n_h_*) | 0.033 | 0.233 | 0.200 |
| Per Capita Hunting Rate (*h*) | 0.066 | 0.233 | 0.167 |
| Prey ^14^C Sample Size (*n_a_*) | 0.033 | 0.167 | 0.133 |
| Max. Prey Annual Pop. Growth Rate (r*_a_*) | 0.066 | 0.133 | 0.067 |
| Founding Human Pop. Size (*H_0_*) | 0.100 | 0.133 | 0.033 |
| Colonization Year in yr BP | 0.100 | 0.133 | 0.033 |

*Table S7. Results of a multiple linear regression analysis examining the input parameters on standardized median overlap rate. The table reports unstandardized regression coefficients (Estimate), standard errors, t-statistics, p-values, significance levels, and partial coefficients of determination (partial r²).*

| **Variable** | **Estimate** | **Std Error** | **t** | **Partial r^2^** | **p** |
| --- | --- | --- | --- | --- | --- |
| **Founding Human Pop. Size (*H_0_*)** | **0.000** | **0.000** | **-5.55** | **0.003** | **<<0.001***** |
| **Max. Human Annual Pop. Growth Rate (r*_h_*)** | **-12.116** | **0.164** | **-74.09** | **0.357** | **<<0.001***** |
| **Human Carrying Capacity (K*_h_*)** | **0.000** | **0.000** | **-54.21** | **0.229** | **<<0.001***** |
| **Max. Prey Annual Pop. Growth Rate (r*_a_*)** | **0.989** | **0.038** | **26.00** | **0.064** | **<<0.001***** |
| **Prey Carrying Capacity (*K_a_*)** | **0.000** | **0.000** | **73.06** | **0.35** | **<<0.001***** |
| **Per Capita Hunting Rate (*h*)** | **-0.183** | **0.004** | **-48.44** | **0.192** | **<<0.001***** |
| Colonization Year in yr BP | 0.000 | 0.000 | -1.82 | 0 | 0.07 |
| **Prey ^14^C Sample Size (*n_a_*)** | **0.001** | **0.000** | **30.49** | **0.086** | **<<0.001***** |
| **Archaeological ^14^C Sample Size (*n_h_*)** | **0.003** | **0.000** | **61.37** | **0.276** | **<<0.001***** |
| *Note: Lines in bold face denote a significant result. Significance levels are denoted as follows: * p < 0.05; ** p < 0.01; *** p < 0.001.* | | | | | |

**Time to Extinction**

Time to extinction was influenced primarily by human and prey demographic parameters and was largely insensitive to sampling intensity (Tables S8 and S9). Human population growth rate (*r_h_*) was by far the strongest predictor of extinction time (partial r^2^=0.675) after controlling for all other variables. Increasing human population growth rates (*r_h_*) substantially accelerated extinction, reducing median time to extinction from 646 to 126 years across the range of values examined. Prey carrying capacity (*K_a_*) was the second most important predictor, accounting for 28.0% of the variation and increasing extinction times by approximately 125 years across its tested range. Founding human population size (*H_0_*) and per-capita hunting rate (*h*) also had substantial effects, accounting for 17.8% and 14.5% of the variation, respectively. In contrast, colonization year, archaeological sample size (*n_h_*), and prey radiocarbon sample size (*n_a_*) had little or no effect on extinction times. Overall, these results indicate that extinction dynamics are governed primarily by the balance between human population growth and prey population resilience rather than by the timing of colonization or the intensity of archaeological and paleontological sampling.

*Table S8. Time to extinction in the sensitivity analysis expressed as median minimum, maximum, and range, after aggregation by parameter value.*

| **Input Parameter** | **Min (yr)** | **Max (yr)** | **Range (yr)** |
| --- | --- | --- | --- |
| Max. Human Annual Pop. Growth Rate (r*_h_*) | 126 | 646 | 520 |
| Prey Carrying Capacity (*K_a_*) | 136 | 261 | 125 |
| Founding Human Pop. Size (*H_0_*) | 168 | 261 | 93 |
| Per Capita Hunting Rate (*h*) | 177 | 256 | 79 |
| Max. Prey Annual Pop. Growth Rate (r*_a_*) | 183 | 222 | 39 |
| Human Carrying Capacity (K*_h_*) | 194 | 232 | 38 |
| Colonization Year in yr BP | 201 | 219 | 18 |
| Archaeological ^14^C Sample Size (*n_h_*) | 199 | 213 | 14 |
| Prey ^14^C Sample Size (*n_a_*) | 199 | 212 | 13 |

*Table S9. Results of a multiple linear regression analysis examining the input parameters on standardized median time to extinction. The table reports unstandardized regression coefficients (Estimate), standard errors, t-statistics, p-values, significance levels, and partial coefficients of determination (partial r²).*

| **Variable** | **Estimate** | **Std Error** | **t** | **Partial r^2^** | **p** |
| --- | --- | --- | --- | --- | --- |
| **Founding Human Pop. Size (*H_0_*)** | **-0.002** | **0.000** | **-46.248** | **0.178** | **<<0.001***** |
| **Max. Human Annual Pop. Growth Rate (r*_h_*)** | **-91.965** | **0.641** | **-143.49** | **0.675** | **<<0.001***** |
| **Human Carrying Capacity (K*_h_*)** | **0.000** | **0.000** | **-13.038** | **0.017** | **<<0.001***** |
| **Max. Prey Annual Pop. Growth Rate (r*_a_*)** | **3.128** | **0.149** | **20.983** | **0.043** | **<<0.001***** |
| **Prey Carrying Capacity (*K_a_*)** | **0.000** | **0.000** | **62.088** | **0.280** | **<<0.001***** |
| **Per Capita Hunting Rate (*h*)** | **-0.606** | **0.015** | **-40.927** | **0.145** | **<<0.001***** |
| Colonization Year in yr BP | 0.000 | 0.000 | 0.293 | 0.000 | 0.769 |
| Prey ^14^C Sample Size (*n_a_*) | 0.000 | 0.000 | 1.695 | 0.000 | 0.090 |
| Archaeological ^14^C Sample Size (*n_h_*) | 0.000 | 0.000 | -1.149 | 0.000 | 0.251 |
| *Note: Lines in bold face denote a significant result. Significance levels are denoted as follows: * p < 0.05; ** p < 0.01; *** p < 0.001.* | | | | | |

**Simulation R Code**

#### Initialization of variables ####

extinct <- FALSE #initialization of variable to store whether extinction has happened or not

EndYr<-10000 #years to run

startYr<-17000 #starting date of simulation (yr)

#Human and Animal Population Parameters

Hp0 <- 100 #founding human pop size

Hr = 0.01 #human pop max annual growth rate

Hk = 10000 #human carrying capacity

Ar <- 0.14 #animal max annual pop growth rate

Ak <- 100000 #animal carrying capacity

#Other Parameters

pch <- 0.75 # per capita hunting rate (animals per person per year)

ColYr <-13500 #colonization year

An.Sample.Size<-10 #sample size of dates on extinct fauna

Human.Sample.Size<-30 #sample size of dates on archaeological sites

yr<-rep(999,startYr-EndYr+1) #initialize output dataframe

HumanPop<-rep(999,startYr-EndYr+1) #initialize output dataframe

PreyPop<-rep(999,startYr-EndYr+1)#initialize output dataframe

out.data<-data.frame(yr,HumanPop,PreyPop) #create a data.frame to save results

out.data[1,1]<- startYr #initialize the first row of the output data.frame

out.data[1,2]<- 0 #initialize the first row of the output data.frame

out.data[1,3]<- Ak #initialize the first row of the output data.frame

#### Population Dynamics and Hunting ####

for (t in 1:(startYr-EndYr)) { #loop to control time

out.data[t + 1, 1] <- startYr - t #save the year of the simulation

if (out.data[t + 1, 1] < ColYr) { #this block does human pop

Hpop <- out.data[t , 2] #get the human population for the previous step

r <- (1 - Hpop / Hk) * Hr #calculate the human population growth rate

out.data[t + 1, 2] <- out.data[t, 2] * (r + 1) # save new human pop size

}

if (out.data[t + 1, 1] == ColYr) {out.data[t + 1, 2] <- Hp0} #checks if the colonization year has happened. if so, enter founding pop

if (out.data[t + 1, 1] > ColYr) {out.data[t + 1, 2] <- 0} #if colonization has not happened, human pop is set to zero

Apop <- out.data[t,3] #this does animal population dynamics. the last line includes hunting

r <- (1 - Apop / Ak) * Ar #gets the growth rate for the animal population

if ((out.data[t, 3] * (r + 1) - pch * out.data[t + 1, 2]) < 1) {out.data[t + 1, 3]<-0} else #set animal pop to zero if it falls to less than one

{out.data[t + 1, 3] <- out.data[t, 3] * (r + 1) - pch * out.data[t + 1, 2]} # gets new animal pop if still extant

if (extinct == FALSE & out.data[t + 1, 3] == 0) { #checks if extinct has happened

extinct <- TRUE #if it has happened, set extinct to TRUE

extinct.date<-out.data[t + 1, 1] #saves the extinction date

}

if (extinct==TRUE) {out.data[t+1,3]<-0} #if extinct, set animal population size to zero

}

#### Plot the population trends of humans and prey ####

par(mfrow=c(2,1))

plot(out.data$yr,out.data$HumanPop,type="l",col="palegreen4",ylim=c(0,Ak),xlim=c(17000,10000),ylab="Population Size",xlab="Date (yr)",lwd=2,xaxt="n",yaxt="n",bty="n")

polygon(c(out.data$yr, rev(out.data$yr)),

c(out.data$HumanPop, rep(0, length(out.data$yr))),

col=rgb(102, 139, 63, maxColorValue=255, alpha=100), border=NA)

lines(out.data$yr,out.data$PreyPop,col="deeppink4",lwd=2)

polygon(c(out.data$yr, rev(out.data$yr)),

c(out.data$PreyPop, rep(0, length(out.data$yr))),

col=rgb(139, 10, 80, maxColorValue=255, alpha=100), border=NA)

axis(1,pos=0)

axis(2,pos=17000,las=2)

text(15500,45000,"Prey",cex=1.5,col="deeppink4")

text(11500,25000,"Humans",cex=1.5,col="palegreen4")

#### Taphonomic Modification ####

taph.adj.prey<-out.data[,3]/2.05*10^60*(out.data[,1]+2914)^-14.05 #correct paleontological record using taphonomic curve from surovell and pelton

taph.adj.prey<-taph.adj.prey/max(taph.adj.prey) #standardize to the maximum value

out.data$taph.adj.prey <- taph.adj.prey #save the results in the output data.frame

taph.adj.humans<-out.data[,2]/8589.83*(out.data[,1]+1020.62)^-1.198 #correct archaeological record using taphonomic curve from bluhm and surovell

taph.adj.humans<-taph.adj.humans/max(taph.adj.humans) #standardize to the maximum value

out.data$taph.adj.humans <- taph.adj.humans #save the results in the output data.frame

#### Plot abundance humans and prey in the record over time####

plot(out.data$yr,taph.adj.prey,type="l",col="deeppink4",ylim=c(0,max(taph.adj.prey)),xlim=c(17000,10000),ylab="Relative Abundance",xlab="Date (yr)",lwd=2,xaxt="n",yaxt="n",bty="n")

polygon(c(out.data$yr, rev(out.data$yr)),

c(taph.adj.prey, rep(0, length(out.data$yr))),

col=adjustcolor("deeppink4", alpha.f=0.3), border=NA)

lines(out.data$yr,taph.adj.humans,col="palegreen4",lwd=2)

polygon(c(out.data$yr, rev(out.data$yr)),

c(taph.adj.humans, rep(0, length(out.data$yr))),

col=adjustcolor("palegreen4", alpha.f=0.3), border=NA)

axis(1,pos=0)

axis(2,pos=17000,las=2)

text(15500,0.5,"Prey",cex=1.5,col="deeppink4")

text(11500,0.25,"Humans",cex=1.5,col="palegreen4")

#### Sampling the Record ####

fad<-vector() #create a vector to store first appearance dates

lad<-vector() #create a vector to store last appearance dates

overlaps<-0 #create a variable to store the number of iterations in which fauna and archaeological sites overlap in time

iters<-30 #number of iterations over which to sample

par(mfrow=c(1,1)) #make a single plot window

animal.dates<-sample(out.data$yr,size=An.Sample.Size, prob=taph.adj.prey) #sample the paleontological record

animal.dates

human.dates<-sample(out.data$yr,size=Human.Sample.Size, prob=taph.adj.humans) #sample the archaeological record

plot(animal.dates,rep(1,An.Sample.Size),xlim=c(17000,8000),pch=21,bg=rgb(209,59,166,120,max=255),ylim=c(0.5,iters+2),las=1,ylab="Run",xlab="Date (Years Before Present)",yaxt="n",bty="n",xaxt="n") #create a plot of dates on prey

axis(1,at=seq(16000,10000,-2000)) #add the x-axis

points(human.dates,rep(1,Human.Sample.Size),pch=21,bg=rgb(91,177,105,200,max=255)) #add the archaeological dates

text(9500,iters+1.5,"Extinction",cex=0.8) #add a text label to sit above LAD age offsets

text(9500,1,min(animal.dates-extinct.date),cex=0.8) #add the first LAD age offset

text(8800,iters+2.5,"Colon-",cex=0.8) #add a text label to sit above FAD age offsets 1/2

text(8800,iters+1.5,"ization",cex=0.8) #add a text label to sit above FAD age offsets 2/2

text(8800,1,ColYr-max(human.dates),cex=0.8) #add the first FAD age offset

text(8100,iters+1.5,"Overlap",cex=0.8) #add a text label to sit above stars indicating overlapping records

fad<-append(fad,max(human.dates)) #save the first FAD

lad<-append(lad,min(animal.dates)) #save the first LAD

if (min(animal.dates)<max(human.dates)) {#text(8100,1,"*",cex=2) #if the sampled records overlap, add a star to the first line

overlaps<-overlaps+1} #increment the overlap variable

for (x in 2:iters){ #continue random sampling

animal.dates<-sample(out.data$yr,size=An.Sample.Size, prob=taph.adj.prey) #sample the paleontological record

human.dates<-sample(out.data$yr,size=Human.Sample.Size, prob=taph.adj.humans) #sample the archaeological record

points(animal.dates,rep(x,An.Sample.Size),pch=21,bg=rgb(209,59,166,120,max=255)) #add the paleontological dates

points(human.dates,rep(x,Human.Sample.Size),pch=21,bg=rgb(91,177,105,200,max=255)) #add the archaeological dates

text(9500,x,min(animal.dates-extinct.date),cex=0.8) #add the LAD age offset

text(8800,x,ColYr-max(human.dates),cex=0.8) #add the FAD age offset

if (min(animal.dates)<max(human.dates)) {#text(8100,x,"*",cex=2) #add a star if the records overlap in time

overlaps<-overlaps+1} #increment the overlap variable

fad<-append(fad,max(human.dates)) #save the first appearance date

lad<-append(lad,min(animal.dates)) #save the last appearance date

}

lines(x=c(extinct.date,extinct.date),y=c(0,iters+1),col=rgb(209,59,166,max=255),lwd=2) #add a vertical line to denote when extinction happens

lines(x=c(ColYr,ColYr),y=c(0,iters+1),col="black",lwd=2) #add a vertical line to denote when colonization happens

text(ColYr,iters+1.5,"Colonization",pos=2,cex=0.8) #add a label above the colonization line

text(extinct.date,iters+1.5,"Extinction",pos=4,cex=0.8,col=rgb(209,59,166,max=255)) #add a label above the extinction line

**Sensitivity Analysis R Code**

n.runs <- 10000 #number of runs for sensitivity analysis

#### Initialization of variables ####

extinct <- FALSE #initialization of variable to store whether extinction has happened or not

EndYr<-10000 #years to run

startYr<-20000 #starting date of simulation (yr)

#Human and Animal Population Parameters

Hp0 <- 100 #founding human pop size

Hr = 0.01 #human pop max annual growth rate

Hk = 10000 #human carrying capacity

Ar <- 0.14 #animal max annual pop growth rate

Ak <- 100000 #animal carrying capacity

#Other Parameters

pch <- 0.75 # per capita hunting rate (animals per person per year)

ColYr <-13500 #colonization year

An.Sample.Size<-30 #sample size of dates on extinct fauna

Human.Sample.Size<-30 #sample size of dates on archaeological sites

params <- expand.grid( #creates all possible parameter combos within the following ranges

Hp0 = seq(100,500,100),

Hr = seq(0.005, 0.03, .0025),

Hk = seq(10000, 40000,2000),

Ar = seq(0.1,0.2,0.02),

Ak = seq(40000, 200000,40000),

pch = seq(0.6, 1.6, 0.2),

ColYr = seq(22000, 13000, by = -1000),

An.Sample.Size = seq(10,100,10),

Human.Sample.Size = seq(10,100,10)

)

params2 <- params[sample(1:nrow(params), n.runs, replace = FALSE), ]

big.out.data<-data.frame(params2,rep(FALSE,n.runs),rep(0,n.runs),rep(0,n.runs),rep(0,n.runs),rep(0,n.runs),rep(9999,n.runs),rep(9999,n.runs),rep(0,n.runs),rep(0,n.runs),rep(0,n.runs),rep(0,n.runs),rep(0,n.runs))

names(big.out.data)<-column_headers <- c(

"Hp0", "Hr", "Hk", "Ar", "Ak", "pch", "ColYr",

"An.Sample.Size", "Human.Sample.Size", "extinct",

"extinct.date", "overlap.rate", "median.FAD", "mean.FAD", "median.LAD", "mean.LAD",

"median.FAD.error", "mean.FAD.error", "median.LAD.error", "mean.LAD.error", "time.to.extinction")

for (i in 1:n.runs){ #simulation runs for sensitivity analysis

extinct.date <- NA

Hp0 =params2$Hp0[i] #get the starting human population size

Hr = params2$Hr[i] #get the human maximum population growth rate

Hk = params2$Hk[i] #get the human carrying capacity

Ar = params2$Ar[i] #get the prey population growth rate

Ak = params2$Ak[i] #get the prey carrying capacity

pch = params2$pch[i] #get the per capita hunting rate

ColYr = params2$ColYr[i] #get the colonization year

An.Sample.Size = params2$An.Sample.Size[i] #get the prey sample size

Human.Sample.Size = params2$Human.Sample.Size[i] #get the archaeological sample size

extinct<-FALSE

extinct.data<-0

EndYr<-ColYr-3500 #run until 3500 years after human colonization

startYr<-ColYr+3500

yr<-rep(999,startYr-EndYr+1) #initialize output dataframe

HumanPop<-rep(999,startYr-EndYr+1) #initialize output dataframe

PreyPop<-rep(999,startYr-EndYr+1)#initialize output dataframe

out.data<-data.frame(yr,HumanPop,PreyPop) #create a data.frame to save results

out.data[1,1]<- startYr #initialize the first row of the output data.frame

out.data[1,2]<- 0 #initialize the first row of the output data.frame

out.data[1,3]<- Ak #initialize the first row of the output data.frame

#### Population Dynamics and Hunting ####

for (t in 1:(startYr-EndYr)) { #loop to control time

out.data[t + 1, 1] <- startYr - t #save the year of the simulation

if (out.data[t + 1, 1] < ColYr) { #this block does human pop

Hpop <- out.data[t , 2] #get the human population for the previous step

r <- (1 - Hpop / Hk) * Hr #calculate the human population growth rate

out.data[t + 1, 2] <- out.data[t, 2] * (r + 1) # save new human pop size

}

if (out.data[t + 1, 1] == ColYr) {out.data[t + 1, 2] <- Hp0} #checks if the colonization year has happened. if so, enter founding pop

if (out.data[t + 1, 1] > ColYr) {out.data[t + 1, 2] <- 0} #if colonization has not happened, human pop is set to zero

Apop <- out.data[t,3] #this does animal population dynamics. the last line includes hunting

r <- (1 - Apop / Ak) * Ar #gets the growth rate for the animal population

if ((out.data[t, 3] * (r + 1) - pch * out.data[t + 1, 2]) < 1) {out.data[t + 1, 3]<-0} else #set animal pop to zero if it falls to less than one

{out.data[t + 1, 3] <- out.data[t, 3] * (r + 1) - pch * out.data[t + 1, 2]} #gets new animal pop if still extant

if (extinct == FALSE & out.data[t + 1, 3] == 0) { #checks if extinct has happened

extinct <- TRUE #if it has happened, set extinct to TRUE

extinct.date<-out.data[t + 1, 1] #saves the extinction date

}

if (extinct==TRUE) {out.data[t+1,3]<-0} #if extinct, set animal population size to zero

}

#### Plot the population trends of humans and prey ####

par(mfrow=c(2,1))

plot(out.data$yr,out.data$HumanPop,type="l",col="palegreen4",ylim=c(0,Ak),xlim=c(25000,10000),ylab="Population Size",xlab="Date (yr)",lwd=2,xaxt="n",yaxt="n",bty="n")

polygon(c(out.data$yr, rev(out.data$yr)),

c(out.data$HumanPop, rep(0, length(out.data$yr))),

col=rgb(102, 139, 63, maxColorValue=255, alpha=100), border=NA)

lines(out.data$yr,out.data$PreyPop,col="deeppink4",lwd=2)

polygon(c(out.data$yr, rev(out.data$yr)),

c(out.data$PreyPop, rep(0, length(out.data$yr))),

col=rgb(139, 10, 80, maxColorValue=255, alpha=100), border=NA)

axis(1,pos=0)

axis(2,pos=16000,las=2)

text(15500,45000,"Prey",cex=1.5,col="deeppink4")

text(11500,25000,"Humans",cex=1.5,col="palegreen4")

#### Taphonomic Modification ####

taph.adj.prey<-out.data[,3]/2.05*10^60*(out.data[,1]+2914)^-14.05 #correct paleontological record using taphonomic curve from surovell and pelton

taph.adj.prey<-taph.adj.prey/max(taph.adj.prey) #standardize to the maximum value

out.data$taph.adj.prey <- taph.adj.prey #save the results in the output data.frame

taph.adj.humans<-out.data[,2]/8589.83*(out.data[,1]+1020.62)^-1.198 #correct archaeological record using taphonomic curve from surovell and pelton

taph.adj.humans<-taph.adj.humans/max(taph.adj.humans) #standardize to the maximum value

out.data$taph.adj.humans <- taph.adj.humans #save the results in the output data.frame

#### Plot abundance humans and prey in the record over time####

plot(out.data$yr,taph.adj.prey,type="l",col="deeppink4",ylim=c(0,max(taph.adj.prey)),xlim=c(25000,10000),ylab="Relative Abundance",xlab="Date (yr)",lwd=2,xaxt="n",yaxt="n",bty="n")

polygon(c(out.data$yr, rev(out.data$yr)),

c(taph.adj.prey, rep(0, length(out.data$yr))),

col=adjustcolor("deeppink4", alpha.f=0.3), border=NA)

lines(out.data$yr,taph.adj.humans,col="palegreen4",lwd=2)

polygon(c(out.data$yr, rev(out.data$yr)),

c(taph.adj.humans, rep(0, length(out.data$yr))),

col=adjustcolor("palegreen4", alpha.f=0.3), border=NA)

axis(1,pos=0)

axis(2,pos=16000,las=2)

text(15500,0.5,"Prey",cex=1.5,col="deeppink4")

text(11500,0.25,"Humans",cex=1.5,col="palegreen4")

#### Sampling the Record ####

fad<-vector() #create a vector to store first appearance dates

lad<-vector() #create a vector to store last appearance dates

overlaps<-0 #create a variable to store the number of iterations in which fauna and archaeological sites overlap in time

iters<-30 #number of iterations over which to sample

par(mfrow=c(1,1)) #make a single plot window

animal.dates<-sample(out.data$yr,size=An.Sample.Size, prob=taph.adj.prey) #sample the paleontological record

human.dates<-sample(out.data$yr,size=Human.Sample.Size, prob=taph.adj.humans) #sample the archaeological record

fad<-append(fad,max(human.dates)) #save the first FAD

lad<-append(lad,min(animal.dates)) #save the first LAD

if (min(animal.dates)<max(human.dates)) {overlaps<-overlaps+1} #increment the overlap variable

for (x in 2:iters){ #continue random sampling

animal.dates<-sample(out.data$yr,size=An.Sample.Size, prob=taph.adj.prey) #sample the paleontological record

human.dates<-sample(out.data$yr,size=Human.Sample.Size, prob=taph.adj.humans) #sample the archaeological record

if (min(animal.dates)<max(human.dates)) {overlaps<-overlaps+1} #increment the overlap variable

fad<-append(fad,max(human.dates)) #save the first appearance date

lad<-append(lad,min(animal.dates)) #save the last appearance date

}

big.out.data$extinct[i] <- extinct #save whether extinction happens

big.out.data$extinct.date[i] <- ifelse(extinct, extinct.date, NA) #save extinction date if extinction happens

big.out.data$overlap.rate[i] <- overlaps / iters #save the overlap rate

big.out.data$median.FAD[i] <- median(fad) #save the median first appearance date

big.out.data$mean.FAD[i] <- mean(fad) #save the mean first appearance date

if (extinct == TRUE) { #if extinction happens

big.out.data$median.LAD[i] <- median(lad) #save the median last appearance date

big.out.data$mean.LAD[i] <- mean(lad) #save the mean last appearance date

big.out.data$median.FAD.error[i] <- ColYr - median(fad) #save the median first appaerance date age offset

big.out.data$mean.FAD.error[i] <- ColYr - mean(fad) #save the mean first appaerance date age offset

big.out.data$median.LAD.error[i] <- median(lad) - extinct.date #save the median last appaerance date age offset

big.out.data$mean.LAD.error[i] <- mean(lad) - extinct.date #save the mean last appaerance date age offset

big.out.data$time.to.extinction[i] <- ColYr - extinct.date #save the time to extinction

} else { #if extinction doesn't happen

big.out.data$median.LAD[i] <- NA #set median LAd to NA

big.out.data$mean.LAD[i] <- NA #set mean LAD to NA

big.out.data$median.FAD.error[i] <- ColYr - median(fad) #save median FAD age offset

big.out.data$mean.FAD.error[i] <- ColYr - mean(fad) #save mean FAD age offset

big.out.data$median.LAD.error[i] <- NA #set median lad age offset to NA

big.out.data$mean.LAD.error[i] <- NA #set mean lad age offset to NA

big.out.data$time.to.extinction[i] <- NA #set extinction time to NA

}

print(i/n.runs)

}

write.csv(big.out.data,"PATHWAY AND FILENAME.csv") #enter a file pathway and file name to output results.
