# Supplementary material for: Why it is easier to date Quaternary extinctions than human colonizations under conditions of overkill
Source: Camb Prism Extinct. 2026 Jul 21;4:e9. doi: 10.1017/ext.2026.10017 (PMC13430543; doi:10.1017/ext.2026.10017)
Supplement: Peer review history

# Reviewer report: Review: Why it is easier to date Quaternary extinctions than human colonizations under conditions of overkill — R0/PR2

**DOI:** 10.1017/ext.2026.10017.pr2

**Authors:** Marco Vidal Cordasco

**Affiliations:** University of Cambridge, United Kingdom of Great Britain and Northern Ireland

This manuscript presents simulation-based research evaluating the extent to which dating errors affect the reliability of establishing chronologies for extinction (LAD) and colonization (FAD) events under an overkill scenario in North America. The core finding (that extinction dating errors are systematically smaller than colonization dating errors) is robust and well supported by the simulations. The study addresses a fundamental issue in Quaternary science, provides testable predictions, and offers valuable insights.

Below I provide several comments that I hope will help improve the paper:

1) As the manuscript states, one of the main reasons for the difference between FAD and LAD is the rapid population collapse caused by hunting. Therefore, it may be appropriate to: a) reflect this explicitly in the title (e.g., Why It Is Easier to Date Quaternary Extinctions than Colonizations under an overkill scenario), or b) to expand the simulations and evaluate different scenarios (comment 2.1 for details)

2) The main limitations arise from several model simplifications: (a) a single species, (b) absence of spatial structure, (c) constant hunting pressure, and (d) limited parameter exploration. Below I comment on what seem to be the most important simplifications, in case the author or editor considers it desirable and feasible to incorporate them into the study:

2.1) The per capita hunting rate (0.75) is held constant regardless of prey density. This could be easily modified either by exploring different hunting rates in the model setup or by allowing hunting pressure to vary as a function of prey density. In addition, exploring different hunting intensities would prevent the analysis from being restricted solely to an overkill scenario.

2.2) Although the model focuses on North America, it is not spatially explicit. Incorporating spatial structure could not only potentially improve realism, but also provide more robust insights (see comment 2.4 for details).

2.3) Human and prey population dynamics are modelled using the Verhulst growth equation. This should be explicitly stated in the Methods section.

2.4.) The carrying capacity (CC) is held constant through time. While this could be improved by incorporating CC estimations derived from fluctuations in Net Primary Productivity (which would allow the simulations to become spatially explicit), I recommend at least exploring several CC values. Many of the reported results (e.g., “… but the earliest archaeological sites typically occur 300 to 600 years after colonization” (lines 52-56)) may be affected by this parameter, so it could be useful to explore different values.

3) The empirical data for the extinct megafauna species used in some of the figures are not provided

4) In lines 39-43: “Again, for the purposes of this paper, I assume extinction by overkill. In the case of a prolonged extinction, one in which a population declines over thousands of years, this may not be true, but I suspect that these kinds of extinctions were rare in the Quaternary period.” I suggest removing the final sentence of this paragraph, as many Quaternary extinctions were not rapid events, at least in Europe (e.g., the extinctions of Stephanorhinus hemitoechus, Ursus spelaeus, or Neanderthals).

5) Based on the results, the author suggests that a sample size of around 30 dates is sufficient to reliably estimate extinction timing. However, obtaining 30 dated bones of a single species, or 30 dated stratigraphic layers, is not a small sample size in practice, especially if the goal is to estimate the LAD or FAD within a relatively small/medium region. For this reason, I suggest the author to further explore the data obtained from the simulations and evaluate the extent to which age-estimation methods, such as Optimal Linear Estimations, can provide reliable extinction estimates with smaller (e.g., 5, 10, 15) sample sizes.

I hope these comments help improve the manuscript or stimulate further development of this modelling approach.

# Reviewer report: Review: Why it is easier to date Quaternary extinctions than human colonizations under conditions of overkill — R0/PR3

**DOI:** 10.1017/ext.2026.10017.pr3

Review for “Why is it Easier to Date Quaternary Extinctions than Colonizations”, by Todd A. Surovell

General comments:

The paper assesses the impact of human colonisation on the extinction of species inhabiting those regions at the time of colonisation. To do this, the model simulates a Lotka-Volterra interaction (limited to a single species), while taking into account possible confounding elements, such as differences in record preservation (e.g. taphonomy), which produce errors in our estimation of last appearance dates (LADs) and first appearance dates (FADs). Arguably, these errors could be hindering potential interaction processes by increasing the error between the recovered latest/earliest date and the actual LAD/FAD.

I must say that I have read this paper with great interest, and that I agree with most of its fundamental theoretical tenets, and the way they are presented. However, I do think that the paper needs a significant amount of work to make its model (and hence its claims) more solid. My concerns revolve particularly around three points.

- Definition of the model: As it stands, the model is a variation of the well-known Lotka-Volterra family of models. However, some fundamental aspects could be added (perhaps as supplementary information) to clarify how specifically it is applied here. Firstly, the model is not mathematically defined. A mathematical definition would be clearer than a narrative description (and would save space in the text if needed). Secondly, the model needs more exploration to understand and put its outcomes in context. Equilibriums are not computed (or at least not shown). This is a common practice in LV models to understand the model’s theoretical behaviour. More importantly, a sensitivity analysis would be needed to understand the potential impact of the different parameters and their ranges. While parameter value is defined based in previous literature, potentially different parameter combinations could give similar (or different) results. This is not explored in the model, and it would be necessary to assess potential problems of equifinality, which are quite common in this specific research context. (See, for instance, Aoki, 2020; or, less strictly, Cortell-Nicolau et al., 2025).

- Calibration error and sample size: I’m unsure on how the uncertainty on C14 calibration has been treated. The paper mentions that it uses the mean of calibrated ages. I understand this is using each calibrated date as a single point estimate (please, do correct me if I’m wrong). If this is the case, firstly, due to the multimodality and frequent skewness of C14 calibrated distributions, the mean might not be the most appropriate summary statistic, for which the median may be more appropriate. However, even the medians are not optimal, since they do not account for the irregularity and high uncertainty of the calibration on specific points of the calibrated probability mass (think, for instance, on calibration plateaus, but not only). In this regard, resampling processes could be more consistent. This resampling technique, of course, is significantly more expensive computationally speaking than simply using a summary statistic, but is able to account for the uncertainty of the calibration. Given the context here, the estimation of single point estimates is important, and a ‘wrong’ estimation can give spurious results. Hence, perhaps it would be useful to explicitly include the uncertainty on the calibration, rather than simply offer single points which might or might not be biased.

Additionally, the paper uses 30 dates for a time span of several thousand years (the number 3,500 years is mentioned, but the plots offer an interval of 6,000 years). I would consider this an insufficient sample to account for that time range both theoretically and empirically. Empirically speaking, for that time span, several archaeological contexts should have dates on the hundreds. As the author is surely aware of, it has been shown theoretically (see Rhode et al., 2014) how smaller sample sizes can result in artificial peaks, troughs and even gaps in the record. Admittedly, peaks and troughs deserve attention on their own (e.g. Bronk-Ramsey, 2017; Crema & Bevan, 2021) and might not be specifically relevant to this paper. However, gaps are, and with this small dataset it is possible that the estimation errors observed might be related to these gaps, created because of sample size, and not actual population dynamics. This possibility is also enforced by the wide ranges and values offered in figure 2. Therefore, I would strongly suggest to reproduce this model, ideally with different sample size values or, at least, some sample size large enough, and which resembles the sample sizes recovered in real archaeological datasets for this time span.

- No mention of previous work on earliest/latest occurrences: One of the key arguments of the paper is that inaccurate estimates of specific LDAs and FDAs can lead to misleading theoretical interpretations. However, the paper is neglecting a significant line of work both in archaeology and biology dedicated to dealing with this specific problem (see works by Solow, Roberts, Rivadeneira, Key, Brunner and others). It would be interesting to see how this approach relates to that line of work.

Minor comments:

- Line 20 (p. 4): “because were are unlikely to find specimens [...]”. Potential missing word or errata?

- Line 20 (p. 8): The term ‘dating error’ has very specific meaning in the context of C14 dating (e.g. the standard deviation of the calibrated dates). Because of that, it can be confusing to understand what the author specifically refers to here. Would it be possible to find a different wording for ‘dating error’ as it is understood in this paper?

- Line 8 (p. 9): “Expected FADs and LADs both get older”. I understand LADs, but why would FADs change based on population growth rate? Unless a spatial component is considered, which takes into account the demographic saturation at the point of origin, FAD is just the first arrival, so it is independent from growth rate. The author, however, clearly specifies that spatial components are not taken into consideration (lines 13-14 p. 10). Could the author please clarify?

- Figure 1a: The impact shown by humans is very strong even with low population numbers. Perhaps the sensitivity analysis (mentioned above) could help clarify expected impacts given different population sizes and parameter values.

- Figure 1b: Is that (the y-axis) the proportion of relative abundance given the total population of prey/humans? If it is, it might be misleading in the way it is presented. The figure gives the impression of similar amount of remains; however, if we consider the actual population of each group (1a), the amount of remains should be clearly different. A potential legitimate counterargument to this is that in lines 31-39 (p. 11). If this is the case, it would be useful that the author clearly stated that it is, and how is this differential preservation due to the production of artefacts by humans has been taken into account.

- Figure 4b: See my comment above re the impact of growth rate on FADs.

References

Aoki, K. (2020). A three-population wave-of-advance model for the European early Neolithic. PLOS ONE, 15(5), e0233184. https://doi.org/10.1371/journal.pone.0233184

Bronk-Ramsey, C. (2017). Methods for Summarizing Radiocarbon Datasets. Radiocarbon, 59(6), 1809–1833. https://doi.org/10.1017/RDC.2017.108

Cortell-Nicolau, A., Rivas, J., Crema, E. R., Shennan, S., García-Puchol, O., Kolář, J., Staniuk, R., & Timpson, A. (2025). Demographic interactions between the last hunter-gatherers and the first farmers. Proceedings of the National Academy of Sciences, 122(14), e2416221122. https://doi.org/10.1073/pnas.2416221122

Crema, E. R., & Bevan, A. (2021). Inference from large sets of radiocarbon dates: Software and methods. Radiocarbon, 63(1), 23–39. https://doi.org/10.1017/RDC.2020.95

Rhode, D., Brantingham, P. J., Perreault, C., & Madsen, D. B. (2014). Mind the gaps: Testing for hiatuses in regional radiocarbon date sequences. Journal of Archaeological Science, 52, 567–577. https://doi.org/10.1016/j.jas.2014.02.022

# Editor report: Recommendation: Why it is easier to date Quaternary extinctions than human colonizations under conditions of overkill — R0/PR4

**DOI:** 10.1017/ext.2026.10017.pr4

**Affiliations:** University of Cambridge, United Kingdom of Great Britain and Northern Ireland

Thanks are extended to the author for their interesting submission. Both reviewers were positive about the manuscript, finding it to be important and publishable subject to revision. A ‘major’ revision recommendation is returned based on the need for some additional modelling. But all suggested changes are straightforward, albeit they may require some limited additional coding. R2 raises two important points concerning how the model is defined, and how calibration error and samples size are dealt with. Both are important and require the author to revise the manuscript, and slightly alter their analyses to more fully explore the issues at hand. R1 also raises interesting scenario changes and model re-runs to more fully explain the results in the present set of analyses. A few points were shared between the reviewers (engagement with alternative methods and sample size) and I agree revising in response to these points would be helpful. A few additional comments have been provided by both reviewers; all appear measured and directed towards strengthening the interesting results. If the author declines to revise in response to any of the reviewer comments it would be appreciated if they could provide a detailed explanation.

# Editor report: Decision: Why it is easier to date Quaternary extinctions than human colonizations under conditions of overkill — R0/PR5

**DOI:** 10.1017/ext.2026.10017.pr5

**Affiliations:** School of Natural Sciences, University of Tasmania, Australia

No accompanying comment.

# Reviewer report: Review: Why it is easier to date Quaternary extinctions than human colonizations under conditions of overkill — R1/PR7

**DOI:** 10.1017/ext.2026.10017.pr7

**Authors:** Marco Vidal Cordasco

**Affiliations:** University of Cambridge, United Kingdom of Great Britain and Northern Ireland

I thank the editor for the opportunity to review the revised version of the manuscript, and I appreciate the author’s efforts in addressing the main concerns raised earlier. In my view, the manuscript is suitable for publication, as all major issues have been resolved.

My only remaining concern relates to the data for extinct megafauna species used in several figures, which are not currently provided. This raises an issue regarding the reproducibility of the results. I understand the author’s point that these data have already been published elsewhere and that appropriate references have been cited. However, since the author has already invested effort in compiling these data, making them openly available (while giving full credit and citation to the original sources, of course) would represent a valuable contribution to the scientific community. This would also enhance, in my opinion, the transparency and impact of the paper.

If the dataset is too large to be included in the Supplementary Materials, an alternative would be to deposit it in a public repository such as Zenodo or OSF and provide a DOI link. In addition to this, I have no further concerns and commend the author for the work on this manuscript.

# Editor report: Recommendation: Why it is easier to date Quaternary extinctions than human colonizations under conditions of overkill — R1/PR8

**DOI:** 10.1017/ext.2026.10017.pr8

**Affiliations:** University of Cambridge, United Kingdom of Great Britain and Northern Ireland

Thanks are extended to the author for the substantial work that went into the revised manuscript. Both reviewers were invited to re-review the manuscript, but only one was available to do so. On the basis of their comments and my own reading of the manuscript I consider it publishable, but would invite the author to respond to the reviewer’s final point concerning data availability. 

Additional minor points that may as well be addressed at this stage, since the manuscript is being returned:

The word colonization is used here exclusively with reference to humans, and extinction is used exclusively with reference to fauna (albeit there is a little discussion of human population extirpation towards the end). But, of course, fauna can colonize and humans can go extinct. Predators other than humans can also create overkill scenarios. The author may want to consider if the title would benefit from the inclusion of ‘human’, to clarify the manuscript’s focus. Perhaps ‘human colonization’ or ‘human overkill’?

Page 5 line 31 – ‘…post-date its initial evolution…’. Perhaps rephrase, as ‘initial evolution’ is a biologically vague term given we mostly draw lines in the sand as and when new phenotypes appear in the fossil record. Additionally, the arrival of a species in a new region would have a similar impact (as you state later in term of humans).

Page 5 line 43 – ‘…site created by humans’. It could be helpful to clarify that you mean in North America.

Page 6 line 33 – ‘…provided in the supplementary information.’?

# Editor report: Decision: Why it is easier to date Quaternary extinctions than human colonizations under conditions of overkill — R1/PR9

**DOI:** 10.1017/ext.2026.10017.pr9

**Affiliations:** School of Natural Sciences, University of Tasmania, Australia

No accompanying comment.

# Editor report: Recommendation: Why it is easier to date Quaternary extinctions than human colonizations under conditions of overkill — R2/PR11

**DOI:** 10.1017/ext.2026.10017.pr11

**Affiliations:** University of Cambridge, United Kingdom of Great Britain and Northern Ireland

All changes have been made and the article can be moved to production.

# Editor report: Decision: Why it is easier to date Quaternary extinctions than human colonizations under conditions of overkill — R2/PR12

**DOI:** 10.1017/ext.2026.10017.pr12

**Affiliations:** School of Natural Sciences, University of Tasmania, Australia

No accompanying comment.
